# Fatty acid isotopic composition in Atlantic pollock is not influenced by environmentally relevant dietary fat concentrations

**DOI:** 10.1007/s00442-023-05403-z

**Published:** 2023-06-30

**Authors:** Suzanne M. Budge, Kathryn Townsend, Susan E. Ziegler, Santosh P. Lall

**Affiliations:** 1 Department of Process Engineering and Applied Science, Dalhousie University, Halifax, NS B3H 4R2 Canada; 2 Department of Biology, Dalhousie University, Halifax, NS B3H 4R2 Canada; 3 Department of Earth Science, Memorial University of Newfoundland, St. John’s, NF A1B 3X5 Canada; 4 Department Animal Science and Aquaculture, Faculty of Agriculture, Dalhousie University, Truro, NS B2N 5E3 Canada

**Keywords:** Compound-specific isotopic analysis, Trophic enrichment factor, Lipid

## Abstract

The application of fatty acid (FA) isotopic analysis has great potential in elucidating food web structure, but it has not experienced the same wide-spread use as amino acid isotopic analyses. The failure to adopt FA isotopic methods is almost certainly linked to a lack of reliable information on trophic fractionation of FA, particularly in higher predators. In this work, we attempt to address this shortfall, through comparison of FA δ^13^C values in captive Atlantic pollock (*Pollachius virens*) liver and their known diets. Since catabolism is likely the main cause of fractionation and it may vary with dietary fat content, we investigated the impact of dietary fat concentration on isotopic discrimination in FA. We fed Atlantic pollock three formulated diets with similar FA isotopic compositions but different fat concentrations (5–9% of diet), representative of the range found in natural prey, for 20 weeks. At the conclusion of the study, δ^13^C values of liver FA were very similar to the FA within the corresponding diets, with most discrimination factors < 1. For all FA except 22:6n-3, dietary fat had no effect on discrimination factors. Only for 22:6n-3 did fish fed the highest fat diet have lower δ^13^C values than the diet consumed. Thus, these FA-specific discrimination factors can be applied to evaluate diets in marine fish consuming natural diets and will serve as additional and valuable biomarkers in fish feeding ecology.

**Supplementary Information:**

The online version contains supplementary material available at 10.1007/s00442-023-05403-z.

## Introduction

Bulk stable isotope analysis has been used in numerous applications to provide information about trophic interactions, foraging ecology, and predator diets (Fry 1988; Hobson and Clark 1992; Bloomfield et al. 2011; Layman et al. 2012). The use of naturally occurring stable isotope ratios in ecological applications relies on the premise that their values are predictably changed as they are transferred to consumers at higher trophic levels. Thus, if prey items, or groups of prey, have distinct isotopic ratios, those ratios can be recognized in the predators’ tissues. With representative sampling of predator and potential prey, mixing models (e.g., Fry and Sherr 1984; Phillips and Greg 2003; Parnell et al. 2010) can then be used to determine likely contributions of prey that would yield the isotopic patterns found in the predators’ tissues. Consumer diets have been investigated using stable isotopes of carbon and nitrogen in a wide range of organisms from top predators, such as sharks and seals (Rau et al. 1992; Tucker et al. 2007; Carlisle et al. 2012), to herbivores utilizing different plant sources (Boutton et al. 1983; Codron et al. 2007; Radloff et al. 2013); hydrogen and oxygen stable isotopes are also becoming more commonly applied in food web studies (reviewed in Zanden et al. 2016).

Despite their widespread application, there are several inherent difficulties associated with the use of bulk stable isotopes. For instance, many studies measure only two isotopes (typically C and N) yet strive to attribute many more than three sources to diet, creating an underdetermined system without a single solution. This has led to the development of a variety of approaches (e.g., Phillips et al. 2005; Parnell et al. 2010) but challenges will persist in systems where sources share very similar δ^13^C and δ^15^N values and therefore cannot be distinguished as unique dietary items. With compound-specific analysis, sources can often be assigned unique isotopic fingerprints (Larsen et al. 2009), avoiding these issues and providing insight into complex ecosystems (see Whiteman et al. 2019 for a review).

A second difficulty involves isotopic routing during deposition in the tissue, when the isotopes of the different dietary nutrients are routed to specific tissues (Gannes et al. 1997). For instance, carbon from dietary protein is much more likely to be incorporated into muscle tissue than carbon from dietary carbohydrate. Thus, if muscle tissue containing protein is sampled in an omnivore, the contribution of plants (mainly carbohydrate) in the diet may be underestimated. With bulk isotopes, appropriate selection of sampling tissue may avoid some of the problems associated with routing (Gannes et al. 1997) but it is often an unacknowledged issue.

Finally, isotopic fractionation may occur when a reaction involves forming or breaking a bond that contains the elements of interest; chemical kinetics dictate that the molecule with the lighter isotope participating in the bond breaking or formation will react at a faster rate than one with a heavier isotope at that position so that the products of the reaction have a different distribution of heavy and light isotopes than the reactants (Bigeleisen 1965; Fry 2006). In animals, predator tissues are usually enriched in ^13^C, showing an increase in bulk δ^13^C by ~ 1 ‰ relative to diet, due to the preferential release of ^12^CO_2_ during catabolism of the different tissue types (DeNiro and Epstein 1978). A similar effect is observed with nitrogen, with preferential excretion of the lighter isotope, ^14^N, in urea and ammonia so that the consumer becomes enriched in the heavier isotope ^15^N, by ~ 3 ‰ (Minigawa and Wada 1984). These enrichments of ^13^C and ^15^N, expressed as trophic enrichment or discrimination factors (DF), are incorporated into mixing models and used to estimate predator diets and trophic levels (Gannes et al. 1997). However, numerous studies have demonstrated that DF in the predator are not fixed, and vary among species, tissues within a single species, and diets (DeNiro and Epstein 1978; Caut et al. 2009; Hussey et al. 2014).

Compound-specific isotope analysis (CSIA) has been suggested as an alternative to avoid the limitations associated with bulk isotope analysis (Chamberlain et al. 2006; McMahon et al. 2010; Whiteman et al. 2019). The primary advantage of CSIA over bulk stable isotope analysis is the elimination of issues associated with isotopic routing because the same diet-derived compounds are compared in predator tissue and diet, thus avoiding the problem of differential assimilation of dietary components. Most CSIA applications to date have focused on amino acids (Whiteman et al. 2019), with far fewer reports measuring δ^13^C of fatty acids. However, like some amino acids, several FA are essential, and cannot be synthesized by vertebrates; these essential dietary FA are routed and preferentially stored in fatty tissues in the predator, where they can be easily sampled (Twining et al. 2020). Furthermore, specific FA can be used as biomarkers of plant and algal sources (Dalsgaard et al. 2003) so δ^13^C values of marker FA may be particularly useful in elucidating the structure of complex food webs. Thus, while less commonly applied than AA, FA also show great promise in CSIA applications.

While CSIA offers advantages over bulk analysis in terms of routing, variation between δ^13^C in predator and diet due to fractionation may still exist. When evaluating individual molecules, the metabolic processes leading to fractionation can be much more closely evaluated. Turchini et al. (2022) reviewed the major biochemical transformations that lipids undergo in fish during and after assimilation into tissues. For instance, during digestion, FA are hydrolyzed from their glycerol backbone to cross the intestinal wall and re-esterifed into chylomicrons for distribution to tissues where they may be stored or catabolized for energy. Similar hydrolysis and re-esterification steps occur during mobilization of stored lipids. De novo synthesis of FA also takes place in the liver, as does desaturation and elongation of existing FA. While all of these processes may result in fractionation of C isotopes, our previous work did not find evidence of such fractionation during either digestion of FA or their mobilization from liver into blood, eliminating enzymatically-catalyzed hydrolysis and esterification of FA as a cause of fractionation (Budge et al. 2016). Because marine fish, such as pollock, cannot effectively synthesize polyunsaturated long chain essential FA (Turchini et al. 2009; Tocher 2010), we can also eliminate the contributions of de novo synthesis, desaturation and elongation to fractionation if we target those essential compounds. Thus, the only process that can lead to fractionation is catabolism (or β-oxidation), which uses both saturated and polyunsaturated FA as substrates. Catabolism is expected to target the isotopically lighter FA (with lowest δ^13^C value), leaving FA with higher δ^13^C values in tissues (Deniro and Epstein 1978). In our earlier work (Budge et al. 2016), the similarity in the δ^13^C of essential FA in liver of fed and fasted fish suggested that catabolism was unlikely to lead to fractionation, but, because of prior feeding history, we were unable to calculate reliable diet-to-liver DF.

A number of studies have indicated that the extent of catabolism, and therefore fractionation arising from it, may vary with dietary fat and FA concentration. For instance, Bell et al. (2003) noted that FA present in high concentrations in dietary lipid seemed to be preferentially utilized for metabolism in Atlantic salmon. Similarly, Turchini et al. (2009) suggested that specific FA may be preferentially catabolized and that the extent will vary with dietary content. In contrast, Nanton et al. (2003) measured significantly lower β-oxidation activity in liver tissue of Atlantic haddock (*Melanogrammus aeglefinus*) after feeding a diet composed of 24% lipid compared to 12% lipid, while both Du et al. (2006) and Lu et al. (2014) found a decrease in FA oxidation capacity with feeding of high fat diets in carp (*Ctenopharyngodon idella*) and bream (*Megalobrama amblycephala*). Our recent work also demonstrated different pattens in FA accumulation in liver of Atlantic pollock (*Pollachius virens*) fed diets with different lipid content but similar FA proportions (Budge et al. 2020). Thus, there is no consensus on the influence of dietary FA content on the extent of catabolism; however, variation in that process may have important consequences for empirically determined DF in fish because captive feeding studies often employ commercially-available diets or unique experimental diets (e.g., McMahone et al. 2015; Winter et al. 2019; Franssen et al. 2017; Budge et al. 2016) with fat contents > 10% wwb. Lipid contents of prey of piscivorous fish are usually much lower (1–7% wwb; Budge et al. 2002; Iverson et al. 2002) so it is critical to understand the influence of dietary FA content on DF derived from captive feeding studies.

The overall objective of this project was to establish FA diet-liver DF for fish fed diets with variable fat contents (5–9% wwb). We anticipated that the diets with the highest level of fat would promote greater catabolism of FA than diets with lower fat levels, resulting in DF that varied with dietary fat content. To investigate this, we used Atlantic pollock as a model species, and, in a controlled study, fed the fish one of three diets of different fat content for 20 weeks and then determined δ^13^C in triacylglycerol (TAG) FA in the liver, the primary storage site of FA in lean fish, including pollock (Turchini et al. 2022). The specific objectives were to: (1) evaluate the influence of dietary fat content on δ^13^C of FA in the liver; and (2) calculate diet-to-liver DF for saturated and polyunsaturated FA.

## Materials and methods

### Experimental fish and diets

Adult wild Atlantic pollock were caught using a commercial long line and transferred to the Aquatron Facility at Dalhousie University. Detailed descriptions of animal care, feeds and experimental design can be found in Budge et al. (2020). Briefly, 11 fish were immediately euthanized to serve as initial samples and the remainder were tagged with a passive integrated transponder tag near the dorsal fin. Weight and length were also recorded. They were then separated into 9 tanks with 12 fish in each tank, using tank as the unit of replication (n = 3 each diet). Diets were formulated as moist pellets with lipid contents of ~ 5, 7 and 9% (wwb; referred to as Diets L, M and H, respectively) as described in Budge et al. (2020). After pooling all batches of a diet, six samples of each were taken for determination of lipid concentration and FA profile. Pollock were maintained on one of the three experimental diets for 20 weeks, until their mass had increased ~ three-fold. Each tank of 12 fish was fed to satiation, receiving ~ 40 g of feed twice a day. By the end of 20 weeks, all tanks had 2–4 deaths; all remaining fish were euthanized as described above. From each tank, between 4 and 6 fish were randomly selected, and livers were removed for isotopic analysis.

### Lipid extraction and FA analysis

Whole livers were homogenized separately in a small food processor and diet samples were ground with a mortar and pestle. Lipids were extracted from both sample types following a modified Folch et al. (1957) method and TAG were isolated from the liver samples using thin layer chromatography (Budge et al. 2016). FA methyl esters (FAME) were prepared for all samples using sulfuric acid in methanol as catalyst (Budge et al. 2006). FAME proportions (mass % total FA mass) were determined using a Bruker 436 capillary gas chromatograph (GC) with flame ionization detection in split mode and using a DB-23 column (50%-cyanopropyl-methylpolysiloxane, Agilent; 30 m × 0.25 mm × 0.25 μm) as described in Budge et al. (2006). FA were identified using authentic standards from Nu-Check Prep and by GC-mass spectrometry.

### Isotope analysis

The δ^13^C values of FAME from liver TAG were analyzed on an Agilent 6890N GC coupled to a Thermo Scientific Delta V + isotope ratio mass spectrometer (IRMS) via a ConFlo III interface at the Core Research Equipment and Instrument Training Network (CREAIT Network) at Memorial University in Newfoundland. The inlet was held at 250 °C and 1 μL of sample was injected in splitless mode (purge flow on at 0.5 min) using a GC-PAL A200S autosampler on a BPX-70 column (70% cyanopropyl polysilphenylene-siloxane, SGE Analytical Science; 50 m × 0.32 mm × 0.25 um) with helium as carrier gas at 1.5 mL min^−1^. The initial column temperature was 70 °C, and the temperature was ramped at 10 °C min^−1^ to 160 °C, held for 5 min and then ramped at 4 °C min^−1^ to 260 °C with a 10 min hold. The oxidation reactor was held at 940 °C and was regenerated daily for 10 min. δ^13^C measurements specifically targeted the long-chain PUFA that cannot be synthesized by fish (18:2n-6, 20:5n-3, and 22:6n-3) and FA that were present in proportions > 3% mass of total FA (14:0, 16:0, 16:1n-7, 18:0, 18:1n-9, 18:1n-7, 20:1n-9). FAME were separated by GC and combusted to CO_2_ by a CuO/NiO/Pt oxidation reactor in a Thermo Finnigan GC combustion III interface. Water molecules were removed, and CO_2_ was carried to an IRMS, for analysis of the C isotopes relative to a reference CO_2_ gas that was calibrated against an OzTech CO_2_ standard of − 40.63 ‰ vs. Vienna Peedee Belemnite (VPDB) using the dual inlet method, and had a value of − 40.97 ‰. All δ^13^C values are reported relative to the VPDB standard using standard delta notation. A standard containing 4 FAME and 4 FA ethyl esters of 14:0, 16:0, 18:0, and 20:0 was analyzed several times a day. The certified δ^13^C values were plotted against the measured values, and the equation of the linear regression line was used to make a small daily correction to the measured δ^13^C values of the samples.

An aliquot of the methanol used to methylate FA was analyzed for its δ^13^C composition using an elemental analyzer (Isotope Cube) coupled to a Delta Advantage isotope ratio mass spectrometer (Thermo Fisher). The average δ^13^C of methanol was then used to correct for the additional methyl group added to the FA during transesterification by subtracting the proportional contribution of methanol to the δ^13^C values of FAME with the following equation:

$${\delta }^{13}C=\left(n+1\right)\left[{\delta }^{13}{C}_{FAME}\right]-n[{\delta }^{13}{C}_{FFA}]$$

where n is equal to the number of C atoms in the free FA (FFA). All results were expressed as δ^13^C_FFA_.

### Data and statistical analysis

Tanks were the appropriate unit of replication for testing differences among diets (Hurlbert 1984), so the means of liver TAG FA of the individuals in each tank were calculated (n = 3, each treatment). A Shapiro–Wilk test was used to assess normality of the δ^13^C_FFA_ data of diets, initial liver tissue, and treatment liver tissues. For diets, when normality was confirmed, multivariate analysis of variance (MANOVA) was used to determine if δ^13^C of FA varied according to diet. When MANOVA indicated an overall significant result, ANOVA was performed on each FA variable with a Tukey post-hoc test to determine the similarity of δ^13^C among diets. For the ANOVA, an adjusted p value of 0.005 was used to compensate for multiple testing. Similar approaches were used to evaluate differences in δ^13^C in diets across all FA (ANOVA, followed by Tukey tests) and between the initial and three treatment livers (MANOVA, followed by ANOVA and Tukey tests). Estimated marginal means, and corrected and uncorrected confidence intervals were tabulated for all comparisons (see Supplementary File 1). All raw data derived from the experiments are available in Supplementary File 2.

DF (Δ) were calculated for each FA by subtracting the mean δ^13^C of diet (D) from the mean δ^13^C of treatment liver tissue (T) sample, such that Δ_T-D_ = δ^13^C_T_ − δ^13^C_D_. To determine if the DF were significantly different from zero, MANOVA, followed by ANOVA, was used to compare δ^13^C of the liver and treatment diet for each FA, again using an adjusted p value of 0.005 for the ANOVA. SPSS (Version 26; IBM Corporation; NY, USA) was used for all statistical analysis.

## Results

### Fish growth

All pollock increased in mass and length (relative increases of 220–260%, and 29–29%, respectively) during the experiment (Table 1). The mass-specific growth rate was lower for fish fed diet L than the other two diets (ANOVA:* F*_(2,6)_ = 7.7, *P* = 0.022), while growth rate in terms of length was significantly higher for fish fed diet H (ANOVA: *F*_(2,6)_ = 17.1, *P* = 0.003). Lipid content in the liver also increased during the experiment (Table 1). Final liver lipid content (approx. 50 ± 4% wwb) was significantly higher than the initial liver lipid content (26 ± 10%) for all diets fed (ANOVA: *F*_(3,16)_ = 13.6, *P* < 0.001); however, there was no difference in liver lipid content of fish fed the three diets.

**Table 1:** Mass, length, growth rates and lipid content data in experimental fish fed for 20 weeks (mean ± SE; n = 3)

	Diet L	Diet M	Diet H
Initial mass (g)	357 ± 21	483 ± 16	388 ± 29
Final mass (g)	1119 ± 25	1416 ± 40	1337 ± 73
Growth rate (g d^−1^)	5.4^b^ ± 0.2	6.7 ± 0.2	6.8 ± 0.4
Initial length (cm)	32.4 ± 0.2	35.7 ± 0.5	33.7 ± 0.6
Final length (cm)	41.9 ± 0.4	45.8 ± 0.7	46.6 ± 0.7
Growth rate (mm d^−1^)	0.68 ± 0.04	0.72 ± 0.03	0.92^b^ ± 0.02
Liver lipid content (% wwb)^a^	45.3 ± 2.1	52.7 ± 2.1	52.0 ± 3.7

^a^Initial lipid content was 26 ± 10% wwb

^b^Indicates that the value is significantly different (p < 0.05; Tukey’s Test) than other two measurements in the row

### Experimental diets

**Fig. 1:**
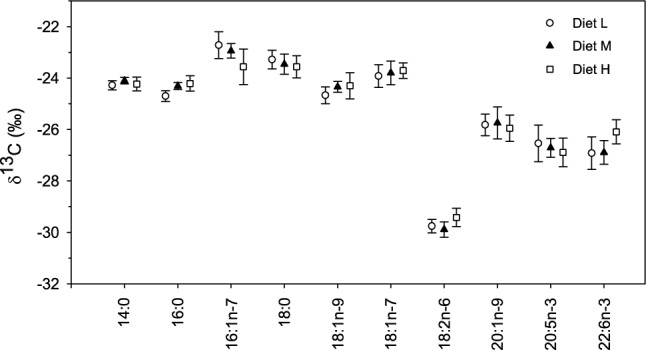
δ^13^C (‰; mean ± SD, n = 6 for each diet) of selected FA in the experimental diets. Within a FA, there was no effect of diet type on δ^13^C value (ANOVA, α = 0.005)

Total fat content (mass % wet weight) increased from diets L to H (4.9, 7.2 and 9.1%), approximately following the proportions formulated for total lipid of 4, 7 and 10%, respectively (Table 1). The FA profiles of the three diets were similar and were dominated by 10 FA (Table 2); only 18:2n-6, 20:1n-9 and 22:1n-11 showed 2 × variation in proportions between diets L and H. The isotopic data showed even greater similarity within each FA, with most FA having < 0.4 ‰ difference among diets (Fig. 1); 16:1n-7 and 22:6n-3 were the exception at ~ 0.8 ‰ difference. Most FA fell into two groups, with δ^13^C values that ranged from − 25 to − 22 ‰ and from − 28 to − 26 ‰; 18:2n-6 did not fall into either group, with δ^13^C values ranging from − 29.6 to − 28.9 ‰. While MANOVA indicated an overall significant multivariate effect of diet type on FA δ^13^C (Wilk’s λ = 0.006, *F*_(20,8)_ = 4.9, *P* =  0.013), univariate tests did not identify any effect of diet type within a FA (α = 0.005; Fig. 1). Comparing mean δ^13^C values across FA, a number were significantly different from each other (ANOVA: *F*_(9,168)_ = 356.8, *P* < 0.005). For example, the mean δ^13^C value for 20:1n-9 was significantly different than all other FA. While mean δ^13^C values for 20:5n-3 and 22:6n-3 were similar, they were both significantly different than all other FA. The FA 18:2n-6 had clearly more negative δ^13^C values than all others. However, 14:0, 16:0, and 18:1n-9 were similar in δ^13^C values, as were 16:1n-7 and 18:0.

**Table 2:** Proportions (mass percent of total FA mass; mean ± SD; n = 6) of the 10 major FA in diets fed to experimental fish

FA	Diet L	Diet M	Diet H
14:0	4.68 ± 0.08	5.33 ± 0.04	5.73 ± 0.04
16:0	17.19 ± 0.46	16.20 ± 0.15	15.42 ± 0.15
16:1n-7	5.41 ± 0.06	6.34 ± 0.04	6.78 ± 0.04
18:0	3.33 ± 0.09	3.13 ± 0.01	3.02 ± 0.02
18:1n-9	10.32 ± 0.09	9.09 ± 0.03	8.70 ± 0.04
18:1n-7	2.98 ± 0.01	2.90 ± 0.01	2.89 ± 0.02
18:2n-6	9.10 ± 0.25	5.60 ± 0.10	3.76 ± 0.13
20:1n-9	2.16 ± 0.03	3.74 ± 0.03	4.69 ± 0.05
20:5n-3	13.21 ± 0.17	13.76 ± 0.09	13.93 ± 0.09
22:6n-3	11.23 ± 0.14	9.92 ± 0.07	9.08 ± 0.07
Total	79.61	76.01	74.00
Fat content	4.9 ± 0.2	7.2 ± 0.2	9.1 ± 0.1

The same FA were also evaluated for δ^13^C values in diet and liver TAG of fish

### Liver TAG FA

FA proportional data for liver TAG FA of these fish were reported in Budge et al. (2020) and are thus only summarized here (Supplementary Table 1). Except for 16:1n-7 and 18:1n-7 in the fish fed Diet L, all FA proportions were substantially different than the initial fish. Proportions of FA in the liver varied with treatment, and roughly mirrored those of the diet; however, 18:1n-7 and 22:6n-3 showed little response to diet, remaining similar in all three treatment groups.

**Fig. 2:**
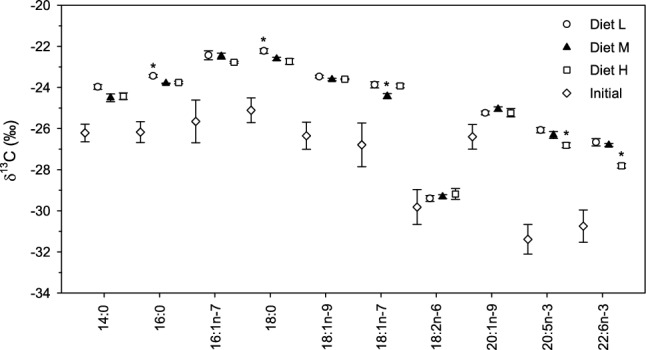
δ^13^C (‰; mean ± SD) for TAG FA in the liver of initial pollock (n = 11) and the liver of fish fed the treatment diets (n = 3 for all). With the exception of 18:2n-6, δ^13^C of all FA in the fish fed the experimental diet were significantly different than in the initial fish. Within a FA, an asterisk indicates that the dietary treatment had a significantly different effect on δ^13^C than the other two experimental diets (Tukey, *P* < 0.05)

Initial liver FA had δ^13^C ranging from − 31 to − 25 ‰, with mean values of 20:5n-3 and 22:6n-3 lowest of all FA (ANOVA: *F*_(9,102)_ = 77.1, *P* < 0.005), while the δ^13^C of FA in treatment liver were generally higher, with the exception of 18:2n-6 (Fig. 2). In the treatment liver, δ^13^C values followed a trend similar to that of diet where most FA had values that fell into two groups, ranging from − 25 to − 22 ‰ or − 28 to − 25 ‰. Also similar to diets, the δ^13^C value of 18:2n-6 was lower than all other FA in the treatment livers. Within a FA, the spread in δ^13^C values according to treatment was larger than that seen with diet, ranging from 0.1 to 1.2 ‰. Mean differences between initial and treatment fish varied from ~ 2 to 5 ‰ for all FA except 18:2n-6 and 20:1n-9 where the mean differences were 0.5 and 1.2 ‰, respectively. δ^13^C values of all FA in the treatment liver, with the exception of 18:2n-6, differed significantly from the δ^13^C values of initial liver (MANOVA: Wilk’s λ = 0.00, *F*_(30,18.3)_ = 8.9, *P* = 0.000005; all univariate ANOVAs: *P* < 0.005; Fig. 2). Within a FA, diet did not affect liver δ^13^C values for 14:0, 16:1n-7, 18:1n-9, 18:2n-6, and 20:1n-9. For 16:0 and 18:0, δ^13^C of Diet L was higher than Diet M and H; for 20:5n-3 and 22:6n-3, δ^13^C of Diet H was lower than the other two. The FA 18:1n-7 was unusual in that Diet M had a lower δ^13^C than Diet L or H (Tukey’s test: *P* < 0.05).

### Discrimination factors

**Fig. 3:**
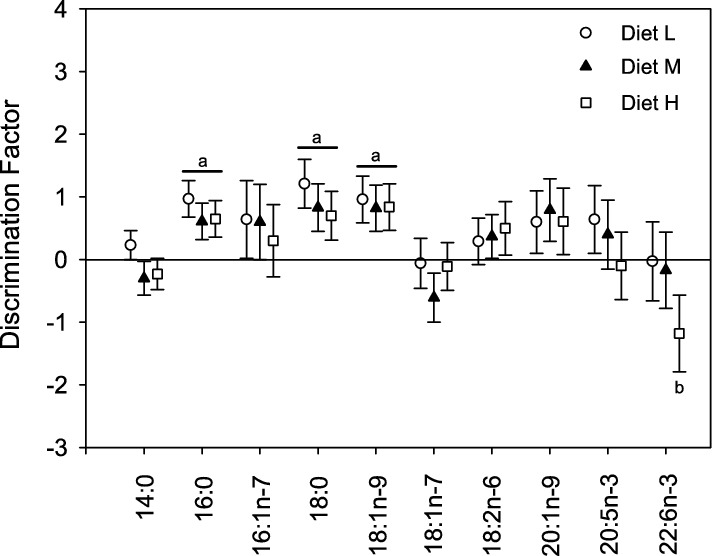
Discrimination factors (Δ; mean ± SD) of FA for liver of pollock fed the experimental diets. The letter ‘a’ indicates that Δ was significantly greater than zero for that FA and all three dietary treatments. A ‘b’ indicates that Δ for the dietary treatment was significantly less than zero for that FA (Tukey, *P* < 0.05)

Since there was no observed effect of fat content on δ^13^C of diet FA, the mean δ^13^C of all diets was used to calculate DF which varied between − 1.2 and 1.2 (Fig. 3). MANOVA indicated that DF were significantly different than zero for all three treatments (Diet L: Wilk’s λ = 0.60, *F*_(10,8)_ = 12.6, *P* = 0.001; Diet M: Wilk’s λ = 0.09, *F*_(10,8)_ = 8.5, *P* = 0.003; Diet H: Wilk’s λ = 0.10, *F*_(10,8)_ = 7.6, *P* = 0.004). Specifically, DF for saturated 16:0 and 18:0 and monounsaturated 18:1n-9 were significantly greater than zero for all three treatments; for 22:6n-3, the DF for the highest fat diet was significantly lower than zero (multiple ANOVA, all with *P* < 0.005). For all other FA, dietary fat content had no influence on DF.

## Discussion

Discrimination factors (DF) are essential in the application of δ^13^C of FA as biomarkers of diet, allowing the δ^13^C value of the predator to be adjusted to reflect fractionation of stable isotopes in the diet. Here we report reliable DF for FA in fish for the first time. Previously (Budge et al. 2016), we lacked confidence in the diet-to-tissue DF that we reported in the same fish species because the fish had been fed a high fat diet for several months before initiating the experiment, leading to the development of liver with lipid content almost 2 × higher than wild fish. Thus, a very long feeding period would be necessary to erase that strong history (Galloway and Budge 2020). In contrast, we are confident that the data in the present study accurately reflect the modifications that fish make to the δ^13^C values of liver FA for two reasons. First, with all diets, fish mass increased by a factor of three or more, and liver lipid content almost doubled. If we assume that liver mass increased by the same factor as whole fish mass and only consider a mechanism where new dietary fat simply ‘dilutes’ the existing FA signature (Robin et al. 2003), ~ 80% of the mass of the liver lipids at the end of the experiment would have been derived from the new diets. Jobling et al. (2008) found that both dilution and turnover occurs in cod tissues, including liver, and the same is likely true in pollock that also primarily stores lipids in the liver. Thus, it is very likely that the tissues had fully responded to the dietary lipids and reflected the effects of fish metabolism on δ^13^C values of FA at the end of the 20-week feeding period. Second, almost all DF were either zero or positive, indicating that the fish tissue δ^13^C value for each FA was the same or higher than the diet; most δ^13^C values of FA in initial fish were several ppt lower than the treatment fish so that treatment fish would have entered the experiment with FA δ^13^C values several ppt less than the diet but ended with those values greater than or equal to diet. This indicates that the fish incorporated and modified the δ^13^C of the diet to a much greater extent than seen in our earlier work (Budge et al. 2016) and we would anticipate that feeding for a longer period would produce little change in the values reported here.

We had expected that fish fed diets with higher fat contents would experience greater catabolism of FA than fish fed lower fat diets because of the excess lipids consumed, leading to higher liver FA δ^13^C values with increasing dietary fat content. However, prior research in this area is conflicting, with empirical studies (e.g., Bell et al. 2003; Turchini et al. 2009) suggesting that FA present at high concentrations are preferentially catabolized, while other work (e.g., Du 2006; Nanton et al. 2003; Lu et al. 2014) that measured activity of enzymes associated with catabolism indicated either a decrease or no difference in hepatic β-oxidation with dietary fat content. Our data support the latter, with increasing dietary fat content having no effect on most FA δ^13^C and resulting in lowest δ^13^C in 16:0, 18:0, 20:5n-3 and 22:6n-3 in fish fed the highest fat diets. The difference in δ^13^C between highest and lowest fat diet in the first three of those four FA was slight (< 0.5 ‰ difference) and may not be biologically significant, particularly when compared to the expected error of ~ 0.5 ‰ introduced by the instrumental analysis (Twining et al. 2020). However, the difference in δ^13^C for 22:6n-3 was > 1 ‰ and the negative DF derived from the highest fat diet indicates that the δ^13^C of that FA in liver is less than diet. This result clearly does not support the idea that increasing dietary fat content leads to higher tissue δ^13^C values.

Classical theory, considering only primary kinetic isotope effects, predicts preferential utilization of molecules with lighter isotopes in reactions that involve the formation and breaking of bonds when there is an excess of substrate. When only considering consumer metabolism, this leads to tissue accumulation of the molecules with heavier isotopes at those positions and, therefore, positive DF (Fry 2006). The same logic applies when considering uptake so that the lowest δ^13^C value of 22:6n-3 found when fish consumed the highest fat diet might be due to preferential uptake of isotopically lighter 22:6n-3, assuming that an excess of that FA was only achieved when the highest fat diet was fed. For this FA, fish fed the low and medium fat content diet had DF of zero, indicating a lack of fractionation, as would be expected if 22:6n-3 was limited in the diet. However, the diets fed here were carefully formulated to meet the minima for essential FA intake in gadoids (National Research Council 2011) so were unlikely limiting even in the lowest fat diets. Moreover, we previously (Budge et al. 2020) fed diets with even higher fat contents (~ 18% wwb), and did not observe fractionation of this FA during hydrolysis and esterification, the only enzymatic processes involved in assimilation of FA into tissues. Thus, it seems unlikely that DF < 0 for 22:6n-3 in fish fed the highest fat diet is simply due to preferential uptake and assimilation of isotopically lighter 22:6n-3.

The FA proportional data was also unusual for 22:6n-3 in that it was one of only two FA that did not respond to the change in dietary FA levels, remaining near constant in all fish. The lack of a direct correlation between diet and tissue proportion for 22:6n-3 is commonly reported; Jardine et al. (2020), in their comparison of dietary FA proportion with animal tissue FA proportions, found 22:6n-3 to be unusual in that in most fish and aquatic invertebrates it seemed to be more efficiently retained in muscle tissue than all other FA and less susceptible to exchange with diet. Here, we were evaluating 22:6n-3 in the liver, functioning as a fat storage site, rather than muscle and would expect tissue FA to better reflect dietary changes. In our earlier work, we interpreted a negative DF to be due to a failure of fish tissues to fully incorporate the FA signal from diet (Budge et al. 2016) due to prior feeding history. With the substantial increase in fish size and liver lipid content recorded over the course of this experiment, we cannot cite the same process. Indeed, we would expect greatest turnover and for DF to approach zero with the highest fat diet, even for a relatively refractory FA. Without a plausible explanation for these unexpected results with the highest fat diet, we can only recommend that dietary fat contents in such captive feeding studies remain as low as possible and certainly ≤ 7% wwb.

Our previous comparisons of δ^13^C of FA in post-prandial and fasted liver (Budge et al. 2016) showed no evidence of β-oxidation of essential FA, supporting our expectations that essential FA would experience little catabolism because they cannot be synthesized de novo (Turchini et al. 2022) and would be routed to fat stores in the liver. This study supports this, with the essential FA 18:2n-6, 20:5n-3 and 22:6n-3 all having DF of zero in the low fat diet, and suggesting that these FA should preserve a dietary δ^13^C signal. Conversely, we had expected that saturated and monounsaturated fatty acids, that can be synthesized by the fish, would have non-zero DF. Our data only partially support this, with 16:0, 18:0 and 18:1n-9 showing DF near 1 ‰, respectively, for the lowest fat diet, while 14:0, 16:1n-7, 18:1n-7 and 20:1n-9 had DF of zero. The FA 16:0 and 18:0 are the two most common products of FA de novo synthesis so the result here could be due to fractionation during synthesis from protein; however, Tocher (2003) indicated that it would be unlikely for marine fish to participate in de novo synthesis because their natural diets are rich in fat. Our lowest fat diet in this work was formulated to mimic the level in natural diet so, from that perspective, all of these fish likely had sufficient fat in their diet to suppress de novo synthesis. Further, we would require a mean δ^13^C value for protein to determine if C isotopes may have been fractionated during FA synthesis and we only measured δ^13^C values for FA. It seems more likely that these non-essential FA may be preferential substrates for oxidation.

Monroig et al. (2018) suggest that the extent of β-oxidation of FA depends on both enzyme specificity and relative FA proportions in the diet (as distinct from total fat content). However, we saw little evidence of an effect of FA proportion. Certainly, the lack of fractionation for essential FA 20:5n-3 and 22:6n-3 in fish fed the lower fat diets points to enzyme specificity dictating the extent of catabolism and the sparing of those FA since they were among those present in highest proportions. For the non-essential saturated and monounsaturated FA, we saw similar fractionation for 16:0, 16:1n-7, 18:0 and 18:1n-9 despite their proportions varying from ~ 3 to 17%, suggesting preferential catabolism of those four FA. Such preferential catabolism of saturated and monounsaturated FA, and sparing of PUFA, has been noted in fish (reviewed in Tocher 2003) and amphipods (Taipale et al. 2021). The similar levels of fractionation for these FA again support the concept that DF are FA-specific.

The initial fish were measured to demonstrate that fish tissues responded to the new diets. They also represent one of very few reports of FA δ^13^C values in fish from the north Atlantic, and fish in general. Our earlier work (Budge et al. 2016) with this species collected similar ‘initial’ samples prior to a diet shift but those were collected after feeding commercial diets for several months in captivity so that their tissues did not represent the δ^13^C FA ratios of wild fish. Here, instead, we have data representing the δ^13^C values of FA in liver of wild fish captured on the Scotian Shelf in the near-shore environment. These δ^13^C values differ distinctively from other studies with marine fish (e.g., Copeman et al. 2009; Graham et al. 2014; Kohlbach et al. 2017), where the δ^13^C values here for the 14, 16 and 18 carbon FA, as well as 20:1n-9, were all consistent (δ^13^C values from − 27 to − 25 ‰) but values for essential PUFA 18:2n-6, 20:5n-3 and 22:6n-3 were much lower (− 32 to − 29 ‰). Prior to this work, the elevated δ^13^C values for the saturated and monounsaturated FA would likely have been interpreted as arising from catabolism of the isotopically-lighter FA (DeNiro and Epstein 1978) and a retention of the essential PUFA; however, our DF show that the initial fish data must reflect a dietary source with lower δ^13^C for 20:5n-3 and 22:6n-3 than that of the saturated and monounsaturated FA. While the literature is inconsistent, some have reported lower δ^13^C values in those PUFA in particulate organic matter (Ramos et al. 2003; Kohlbach et al. 2016), suggesting that the pattern we see here in δ^13^C values of these wild fish was primarily a result of the unique isotopic ratios of primary production on the Scotian Shelf, NS.

In short-term experiments to investigate DF for bulk isotopes or amino acid δ^15^N and δ^13^C, many studies have incorporated a mixture of formulated and natural diets (e.g., McMahone et al. 2015; Colburne et al. 2017; Britton and Busst 2018), with a range of protein contents; the impact of diet quantity on amino acid-specific DF has only recently been demonstrated (Nuche-Pascual et al. 2018), with those authors suggesting, similar to our hypothesis here, that increasing dietary protein content would lead to catabolism and associated fractionation of amino acids. Taipale et al. (2022) pointed out that artificial diets may result in unnaturally high growth rates that can affect FA metabolism and, therefore, fractionation. These studies, combined with our results, clearly highlight the importance of mimicking the composition of natural diets in captive feeding experiments aimed at developing DF for application to wild fish.

Our results describe the first reliable DF for FA in the diet-liver transfer in a marine fish. When fed the lowest fat diet, more similar to natural conditions, fish DF for essential PUFA and most saturated and monounsaturated FA were zero. Three non-essential FA, 16:0, 18:0 and 18:1n-9, did have DF > 1, and our data suggest that enzyme specificity, rather than simply abundance in the diet, led to greater catabolism and thus fractionation of those substrates. For most FA, dietary fat content had no effect on fractionation; however, fish fed the highest fat diet had DF < 0 for the essential FA 22:6n-3. With the application of the DF derived here, we can begin to interpret the δ^13^C values of FA in wild marine fish and incorporate this information into models evaluating their diets.

## Supplementary Information

Below is the link to the electronic supplementary material.

There is a supplementary table of FA proportions (mass % total FA mass) in fish liver (Supplementary Table 1), as well as two supplementary files. (DOCX 15 KB)

Supplementary File 1 tabulates estimated marginal means and both unadjusted and adjusted confidence intervals for all comparisons. (XLSX 29 KB)

All raw data used in the manuscript is available in Supplementary File 2. (XLSX 57 KB)

## Data Availability

The datasets analyzed in the current study are available in the supplementary information.
