# Supplementary material for: Fatty acid isotopic composition in Atlantic pollock is not influenced by environmentally relevant dietary fat concentrations
Source: Oecologia. 2023 Jun 30;202(3):513–22. doi: 10.1007/s00442-023-05403-z (PMC10386935; doi:10.1007/s00442-023-05403-z)
Supplement: Supplementary file 1 — There is a supplementary table of FA proportions (mass % total FA mass) in fish liver (Supplementary Table 1), as well as two supplementary files. (DOCX 15 KB) [file 442_2023_5403_MOESM1_ESM.docx]

**Supplementary Information**

**Fatty acid isotopic composition in Atlantic pollock is not influenced by environmentally relevant dietary fat concentrations**

Suzanne M. Budge^1*^, Kathryn Townsend^2^, Susan E. Ziegler^3^ and Santosh P. Lall^4^

^1^Department of Process Engineering and Applied Science, Dalhousie University, Halifax, NS B3H 4R2, Canada

^2^Department of Biology, Dalhousie University, Halifax, NS, B3H 4R2, Canada

^3^Department of Earth Science, Memorial University of Newfoundland, St. John’s, NF, A1B 3X5, Canada

^4^Department Animal Science and Aquaculture, Faculty of Agriculture, Dalhousie University, Truro, NS, B2N 5E3, Canada

*email: Suzanne.Budge@dal.ca

Table S1. FA proportions (mass % total FA mass) in experimental fish (mean +/- SE, n=3).

|  | Initial | | | Fish (Diet L) | | | Fish (Diet M) | | | Fish (Diet H) | | |
| --- | --- | --- | --- | --- | --- | --- | --- | --- | --- | --- | --- | --- |
| 14:0 | 2.47 | ± | 0.53 | 2.25 | ± | 0.01 | 3.10 | ± | 0.12 | 3.34 | ± | 0.01 |
| 16:0 | 13.88 | ± | 1.45 | 16.35 | ± | 0.13 | 15.74 | ± | 0.21 | 14.79 | ± | 0.17 |
| 16:1n-7 | 4.16 | ± | 1.04 | 4.12 | ± | 0.05 | 5.24 | ± | 0.12 | 5.72 | ± | 0.03 |
| 18:0 | 5.56 | ± | 0.83 | 7.29 | ± | 0.08 | 6.10 | ± | 0.23 | 5.74 | ± | 0.08 |
| 18:1n-9 | 16.08 | ± | 3.01 | 23.70 | ± | 0.22 | 16.93 | ± | 0.78 | 14.94 | ± | 0.00 |
| 18:1n-7 | 4.66 | ± | 0.57 | 4.65 | ± | 0.01 | 4.34 | ± | 0.03 | 4.32 | ± | 0.02 |
| 18:2n-6 | 1.77 | ± | 0.63 | 5.96 | ± | 0.02 | 4.68 | ± | 0.09 | 3.35 | ± | 0.04 |
| 20:1n-9 | 5.58 | ± | 2.06 | 1.92 | ± | 0.01 | 3.51 | ± | 0.05 | 4.35 | ± | 0.01 |
| 20:5n-3 | 5.58 | ± | 2.06 | 10.07 | ± | 0.04 | 12.12 | ± | 0.25 | 13.23 | ± | 0.12 |
| 22:1n-11 | 11.38 | ± | 2.43 | 1.43 | ± | 0.01 | 3.40 | ± | 0.04 | 4.41 | ± | 0.06 |
| 22:6n-3 | 13.74 | ± | 4.51 | 8.42 | ± | 0.07 | 8.66 | ± | 0.14 | 8.70 | ± | 0.07 |
| Total | 84.86 |  |  | 86.18 |  |  | 83.82 |  |  | 82.88 |  |  |
